# Efficient and accurate diagnosis of otomycosis using an ensemble deep-learning model

**DOI:** 10.3389/fmolb.2022.951432

**Published:** 2022-08-19

**Authors:** Chenggang Mao, Aimin Li, Jing Hu, Pengjun Wang, Dan Peng, Juehui Wang, Yi Sun

**Affiliations:** ^1^ Department of Otolaryngology Head and Neck Surgery, Jingzhou Hospital Affiliated to Yangtze University, Jingzhou, Hubei, China; ^2^ Department of Pediatrics, Jingzhou Hospital Affiliated to Yangtze University, Jingzhou, Hubei, China; ^3^ Department of Dermatology, Jingzhou Hospital Affiliated to Yangtze University, Jingzhou, Hubei, China; ^4^ Health Science Center, Yangtze University, Jingzhou, Hubei, China; ^5^ Department of Information Engineering, Jingzhou University, Jingzhou, Hubei, China

**Keywords:** otomycosis, *Aspergillus*, *Candida*, deep-learning, otoendoscopic

## Abstract

Otomycosis accounts for over 15% of cases of external otitis worldwide. It is common in humid regions and Chinese cultures with ear-cleaning custom. *Aspergillus* and *Candida* are the major pathogens causing long-term infection. Early endoscopic and microbiological examinations, performed by otologists and microbiologists, respectively, are important for the appropriate medical treatment of otomycosis. The deep-learning model is a novel automatic diagnostic program that provides quick and accurate diagnoses using a large database of images acquired in clinical settings. The aim of the present study was to introduce a machine-learning model to accurately and quickly diagnose otomycosis caused by *Aspergillus* and *Candida*. We propose a computer-aided decision-making system based on a deep-learning model comprising two subsystems: Java web application and image classification. The web application subsystem provides a user-friendly webpage to collect consulted images and display the calculation results. The image classification subsystem mainly trained neural network models for end-to-end data inference. The end user uploads a few images obtained with the ear endoscope, and the system returns the classification results to the user in the form of category probability values. To accurately diagnose otomycosis, we used otoendoscopic images and fungal culture secretion. Fungal fluorescence, culture, and DNA sequencing were performed to confirm the pathogens *Aspergillus* or *Candida* spp. In addition, impacted cerumen, external otitis, and normal external auditory canal endoscopic images were retained for reference. We merged these four types of images into an otoendoscopic image gallery. To achieve better accuracy and generalization abilities after model-training, we selected 2,182 of approximately 4,000 ear endoscopic images as training samples and 475 as validation samples. After selecting the deep neural network models, we tested the ResNet, SENet, and EfficientNet neural network models with different numbers of layers. Considering the accuracy and operation speed, we finally chose the EfficientNetB6 model, and the probability values of the four categories of otomycosis, impacted cerumen, external otitis, and normal cases were outputted. After multiple model training iterations, the average accuracy of the overall validation sample reached 92.42%. The results suggest that the system could be used as a reference for general practitioners to obtain more accurate diagnoses of otomycosis.

## 1 Introduction

The goal of machine learning is for machines to learn automatically from training data and update their capabilities (Shen et al., 2017). Deep learning is a field of machine learning in which machine learning is implemented using deep neural networks (Giger, 2018). For example, the deep convolutional neural network (CNN) is used by machines to acquire image data and identify the contents in the image (Kooi et al., 2017). Deep learning, or deep neural networks, has been successfully used in some medical fields. For example, in the field of ophthalmology, machine learning can automatically detect retinopathy in patients with diabetes (Horsch et al., 2011; Chougrad et al., 2018).

Otomycosis externa, or fungal otitis externa, is a superficial fungal infection of the external auditory canal that occasionally invades the middle ear. The incidence of otomycosis externa is high in hot and humid climates found in tropical and subtropical regions (Li and He 2019). Patients with this infection usually present with symptoms such as itching, otorrhea, and hearing loss. *Aspergillus* and *Candida* are the most common pathogens causing otomycosis (Kamali Sarwestani et al., 2018). Although otomycosis is rarely fatal, it is difficult to treat because of a long treatment period and easy recurrence. Antifungal drugs show varying sensitivity/resistance to pathogenic bacteria, often leading to poor therapeutic effects (Li et al., 2020). The diagnosis of otomycosis is mainly done clinically by otorhinolaryngologists, and insufficient attention is paid to mycological detection, particularly the subsequent pathogen culture and drug sensitivity test results.

To date, the deep CNN model with various structures has achieved good results in image classification and recognition (Hapfelmeier and Horsch, 2011; Lee et al., 2017). However, the tests were based on standard image galleries and have not been applied in the field of otoendoscopy.

The aim of this study was to establish a comprehensive identification system for otomycosis by comparing the effects of several typical deep CNN models using otoendoscopic images and web applications and selecting three models with the best recognition effects. To diagnose otomycosis accurately, we used otoendoscopic images and fungal culture secretions. The pathogen was identified as *Aspergillus* or *Candida* by fungal fluorescence, culture, and DNA sequencing. In addition, images of impacted cerumen, external otitis, and normal external auditory canal were used as reference to identify otomycosis. We combined these four types of images into a gallery of otoendoscopic images.

## 2 Materials and methods

### 2.1 Design

**FIGURE 1:**
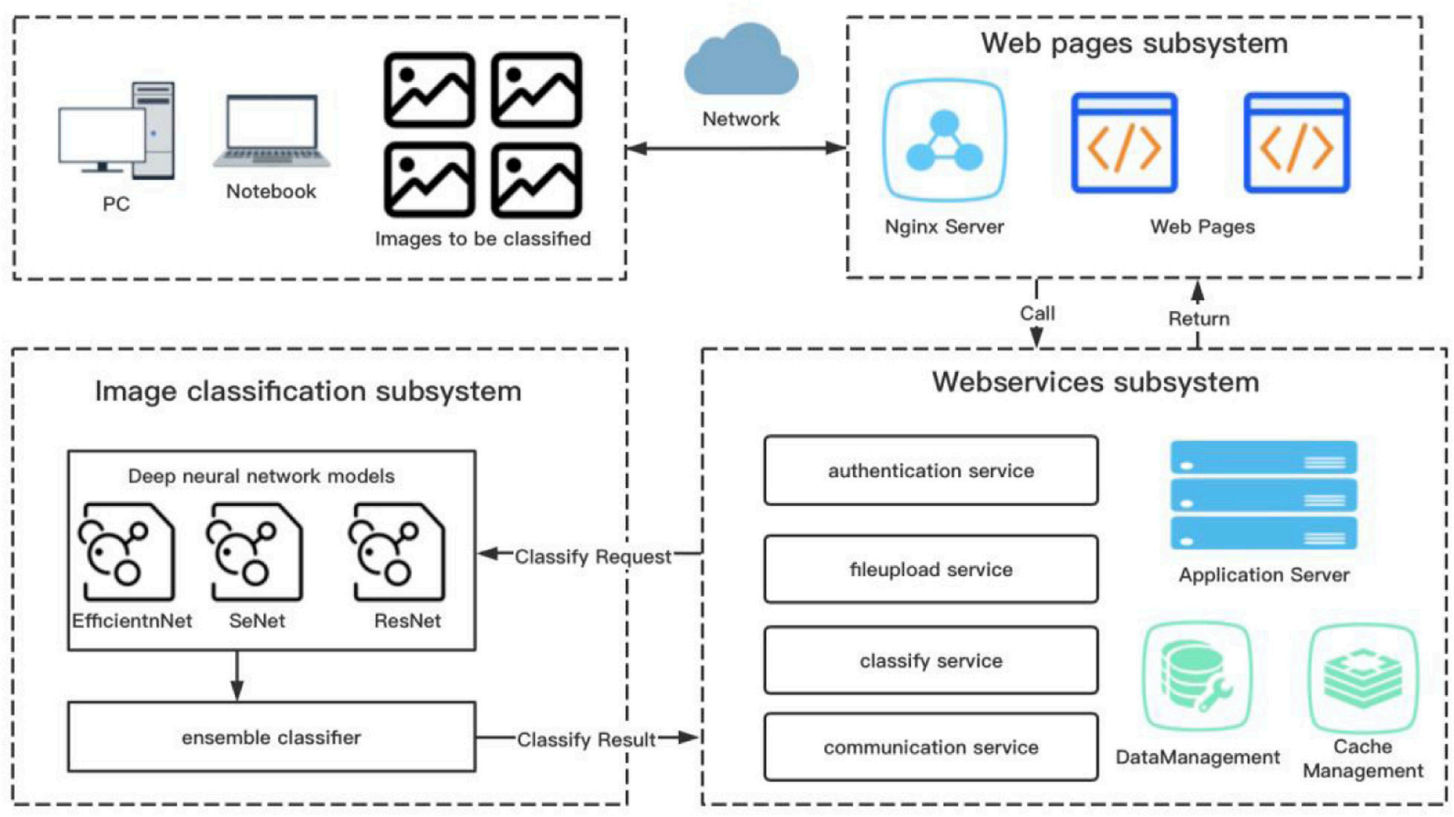
General scheme of the computer-aided system approach to assist the diagnosis of otomycosis.

Figure 1 summarizes the overall design of the web-based computer-aided diagnosis system used in this study. The system comprises three subsystems: front-end page subsystem based on React, business logic subsystem based on SpringBoot, and image classification subsystem based on PyTorch. React is a JavaScript language library for building user interfaces, enabling component-based user interaction pages for easy extension. SpringBoot is a Java language-based framework for quickly building standalone, production-level Java Web services applications. PyTorch is an open-source framework for deep learning based on Python and developed with support from Facebook (Zhang et al., 2019).

According to manufacturer’s instructions, we uploaded a batch of otoendoscopic RGB images up to four at a time to the specific storage space of the server *via* the front-end subsystem. Subsequently, the business logic subsystem verified and cleaned the images. Finally, the image classification subsystem predicted the images for otomycosis, impacted cerumen, external otitis, and the normal external auditory canal. At the end of the model calculation, each image was assigned a percentage of the four categories, adding up to 100%, with the value of each category indicating the probability that the image belonged to that category. Finally, the data were delivered to the front-end page *via* the business subsystem using web services to provide users with diagnostic references. To classify and predict RGB images, we used an end-to-end deep CNN model (Esteva et al., 2017). To train weight parameters, we inputted the classified and labeled training sample images to the model.

### 2.2 Sample

The database used in our study was created at the Department of Otolaryngology - Head and Neck Surgery, Jingzhou Hospital Affiliated to Yangtze University. The study protocol was approved by the Research Ethics Committee of Jingzhou Hospital Affiliated to Yangtze University (protocol number: 2021-093-01). Written informed consent was obtained from patients and caregivers of patients under 18 years of age. All the procedures were carried out in accordance with the tenets of the Declaration of Helsinki.

**FIGURE 2:**
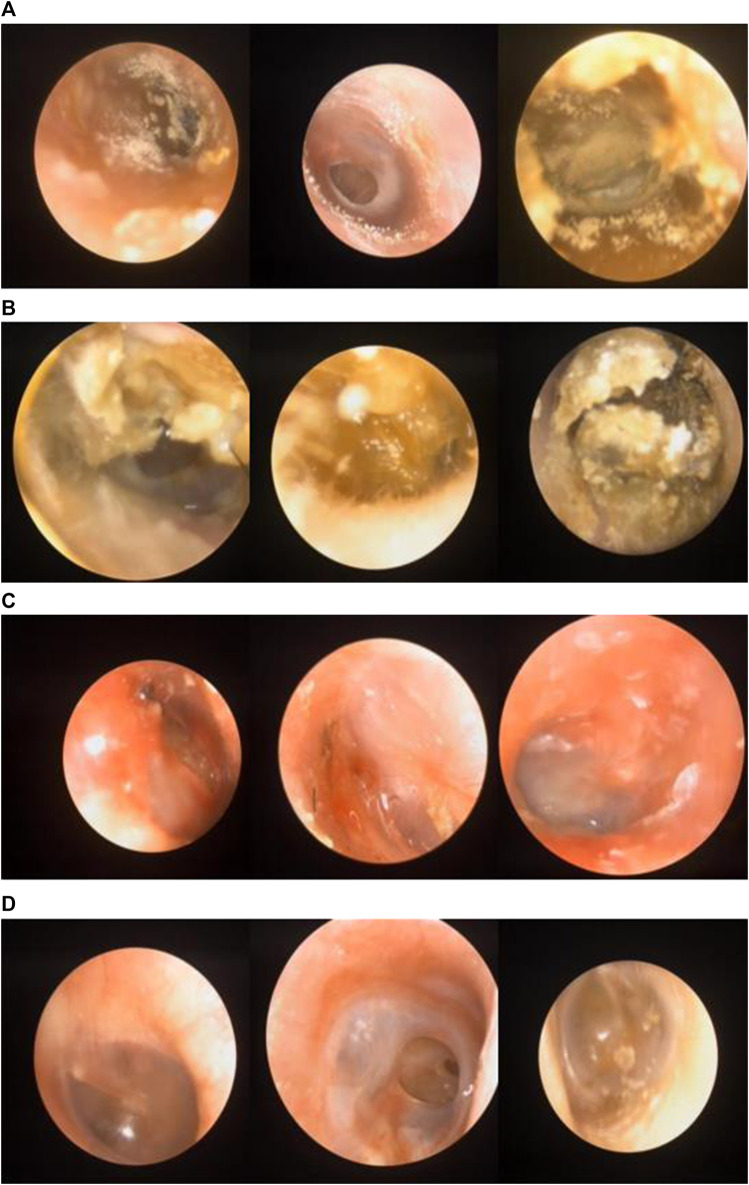
**(A)** Images labeled “otomycosis”. **(B)** Images labeled “impacted cerumen”. **(C)** Images labeled “external otitis”. **(D)** Images labeled “normal case”.

An otorhinolaryngologist evaluated the patients. Patient age ranged from 5 to 72 years. Images with otomycosis, impacted cerumen, external otitis, or the normal external auditory canal were preserved in the otoscopy room. Figures 2A–D shows the representative images of all categories.

To obtain a better training result, we prepared two external auditory canal image datasets: one comprising 2,652 external auditory canal images used to train the model and the other comprising 552 images that were not used to train the model but to test the robustness and accuracy of the model.

### 2.3 Data preprocessing

First, we selected the otoendoscopic RGB image as the system input. The key contents to be identified were in a circular area in the image, while the other areas were black. Due to inter-user differences in operation habits, the circular area varied in size, with the center of the circular area often not coinciding with the center of the image. To maximize the feature data to be recognized by the model for training, we used Hough transform to perform ring detection (Hough ring detection). The center and radius of the circle were detected to calculate the minimum rectangular coordinates. From the rectangular cutting of the original image, excess black boxes were removed (Moses et al., 2018).

Second, because the model was in the training process, the dataset was reused in each iteration of the training. To enhance the robustness of the model, we added the function of random angle rotation to the dataset (Varma and Zisserman, 2009) such that when each sample was removed, the image was rotated randomly.

Third, data standardization was performed. To accelerate the training process, the possibility of the model falling into the local optimum during training was reduced. We treated the input data for data standardization, i.e., according to the image color channel for the unit, the mean and standard deviation values of the training images were calculated. After normalization of each image pixel value, the mean value was subtracted and divided by the standard deviation value. The images with a mean value of 0 and a standard deviation of one were considered normally distributed (Shin et al., 2016). Due to limited memory, 600 training sample otoendoscopic images were randomly selected. The calculated mean value of the RGB channel was [0.5317, 0.3899, 0.3003], while the standard deviation was [0.3482, 0.2748, 0.2329]. The formula for the standard deviation was as follows: $X\;’\;=\;\frac{x-\mu }{\sigma }$

### 2.4 Deep CNN model

The deep CNN model was used in this study. It could analyze the features in its “field of vision” (local receptive field) through neuronal learning of the hidden layer. To enable the CNN model to learn better features from images, many deep CNN models have been proposed (Spasov et al., 2018). We selected three CNN models for training: ResNet, SENet, and EfficientNet (Lee et al., 2020). The algorithm characteristics and design ideas of each model are described below:

#### 2.4.1 ResNet

**FIGURE 3:**
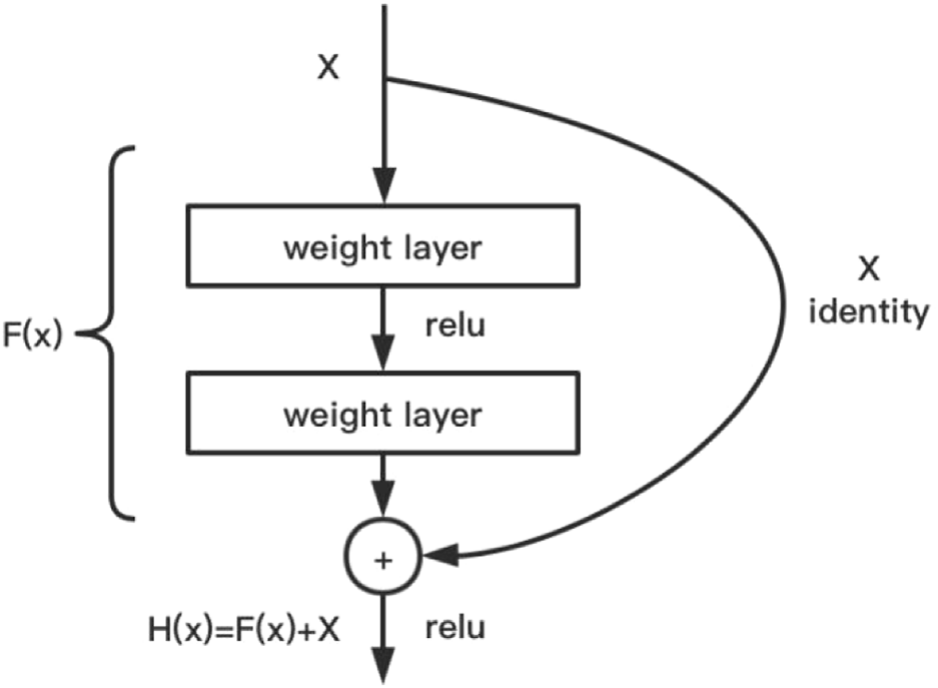
ResNet model.

The main design idea is to introduce a residual network structure (cross-layer jump connection) to resolve SGD optimization difficulties when the neural network model is stacked to a deeper level, and the reverse derivative gradient disappears or explodes, resulting in the deterioration of model performance (Talo et al., 2019). Using the neural network model with a residual structure, the network can be designed deeper, and the training is faster. Because it does not introduce additional parameters or computational complexities and only performs simple addition operation, the computational power consumption is negligible compared to convolution operation. ResNet has designed floors of 18, 34, 50, 101, and 152. Regarding efficiency and cost, ResNet of 101 was used in this study. Figure 3 shows the algorithm and design of this model.

#### 2.4.2 SENet

**FIGURE 4:**
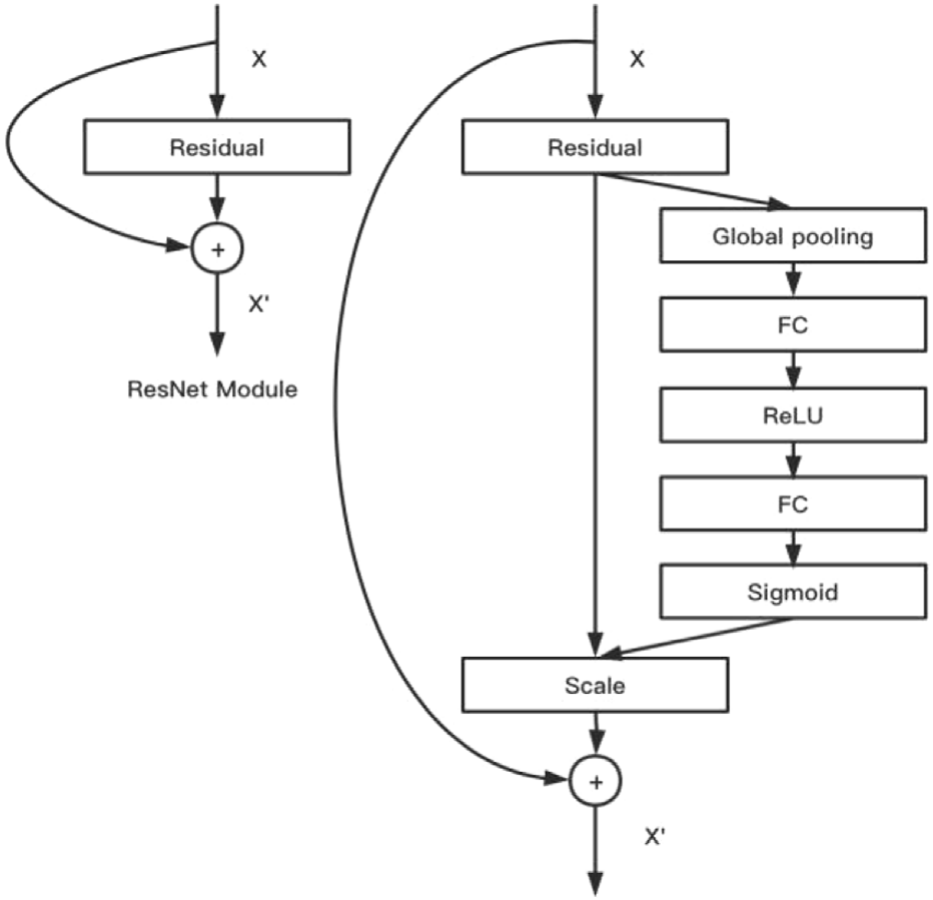
SENet model.

This conventional CNN model aggregates information of image space and feature dimension into a feature channel through the convolution operation of multiple convolution kernels in the local receptive field, and the data of each feature channel are equal (Zhang et al., 2019). However, in real settings, each image is a key feature area, and attention should be paid to the channel data of this part of data conversion. The SE module was designed by the makers of SENet to obtain scaling coefficients (importance) of the channel data through a series of fully connected activation operations of global average pooling and 1 × 1 convolution kernels, which were then weighted to previous features by multiplication. This completed the recalibration of the original feature on the channel dimension. The SE module and ResNet were combined to obtain SENet. In this study, we used the 101-layer SENet. Figure 4 shows the algorithm and design of this model.

#### 2.4.3 EfficientNet

Scaling (model extension) improves the performance of CNN models (Godinez et al., 2017). The expansion direction of the model is mainly divided into the network width (channel, number of convolution kernels), depth (number of layers), and resolution (accuracy of input image); however, these expansions consume abundant additional computing resources. EfficientNetB0 is a simple baseline network with grid structure search. Subsequently, a compound coefficient is used to synthesize the aforementioned dimensions of model extension such that additional consumption of computational power resources can be optimized to improve accuracy. In this study, we used EfficientNetB6.

### 2.5 Deep CNN model training process

#### 2.5.1 Input and output

The size of otoendoscopic RGB images was 515 × 547 pixels, and the standard input size of ResNet101 and SENet101 models was 224 × 224 pixels. Therefore, we added a convolution layer in front of the input layer of the standard ResNet101 and SENet101. The kernel size was 7 × 7. The stride and padding were two and three, respectively, such that the input size of the model became 448 × 448. The input size of the EfficientNetB6 model was 528 × 528. Therefore, the image was scaled to the required size of the model before training. To adapt the output of the model to the objective of our study, the otoendoscopic images were classified into four categories. We replaced the last full connection layer of the standard ResNet101, SENet101, and EfficientNetB6 models with a new full connection layer with four output nodes (Geras et al., 2019).

#### 2.5.2 Optimization of model training

To obtain better training results, we assigned a set of hyperparameters, including whether or not to use pretraining weights, whether or not to carry out center circle interception, batch size, learning rate, and optimizer (Zhang and Suganthan, 2017).

Each time the model was trained, a value was randomly selected from each item. We used the PyTorch framework on two graphics processing units (RTX-2080Ti Gpus). After repeated training of multiple models, we obtained the optimal hyperparameters: using pretraining weight, using center circle interception, batch size of 4, learning rate of 0.001, and SGD optimizer.

### 2.6 Set classifier

Based on the accuracy of the test images, the best training results from among the three models were selected. The average highest accuracies of the ResNet101, SENet101, and EfficientNetB6 models were 78.32, 87.16, and 88.21%, respectively. Table 1 shows the highest accuracies for the four categories. Table 2 shows the weighted mean values of the models for the four categories. The set classifier was obtained by multiplying each model by the weighted mean of each model and category. Table 3 shows the highest accuracy of the final set classifier.

**TABLE 1:** Highest accuracies of the four categories of otoendoscopic images trained by three models.

	Otomycosis (%)	Impacted cerumen (%)	External otitis (%)	Normal case (%)
ResNet101	73.8	78.69	71.19	86.75
SENet101	89.25	78.69	83.33	89.82
EfficientNetB6	95.19	78.69	80.0	86.83

**TABLE 2:** Weighted mean values of the models for four categories of otoendoscopic images.

Weighted mean model	Otomycosis	Impacted cerumen	External otitis	Normal case
ResNet101	0.28578066	0.33333333	0.3035562	0.329347
SENet101	0.34560873	0.33333333	0.35532151	0.34100227
EfficientNetB6	0.3686106	0.33333333	0.3411223	0.32965073

**TABLE 3:** Highest accuracies of four categories of otoendoscopic images by the ensemble classifier.

	Otomycosis (%)	Impacted cerumen (%)	External otitis (%)	Normal case (%)	Average accuracy (%)
Set classifier	94.65	90.16	88.33	92.22	92.42

Evaluation index.

The performance of the set classifier was measured using the obfuscation matrix, precision, recall, precision–recall (PR) curve, and receiver operating characteristic (ROC) curve (Tan et al., 2017).

The confusion matrix represented the number of instances corresponding to the predicted and actual classes. This concept is often used for binary classifications but can be extended to multiclass predictions, with the corresponding class on the diagonal of the matrix and the misclassified class outside the diagonal. We used the set classifier to conduct prediction tests on the verification test set comprising 475 otoendoscopic images. Table 4 shows the confusion matrix.

**TABLE 4:** Confounding matrix results of the test set verified on otoendoscopic images.

Prediction fact	Otomycosis	Impacted cerumen	External otitis	Normal case
Otomycosis	177	3	4	3
Impacted cerumen	2	55	0	4
External otitis	1	0	53	6
Normal case	5	1	7	154

The accuracy refers to the number of predicted positive sample results that are correctly classified. It is calculated using the following formula: precision = true positive/(true positive + false positive). Recall refers to the number of positive sample results that are correctly classified. It is calculated using the following formula: recall = true positive/(true positive + false negative). Table 5 shows the results of the precision and recall.

**TABLE 5:** Accuracy and recall of the four categories of otoendoscopic images.

Indicators	Category			
	Otomycosis (%)	Impacted cerumen (%)	External otitis (%)	Normal case (%)
Precision	95.68	93.22	82.81	92.22
Recall	94.65	90.16	88.33	92.22

**FIGURE 5:**
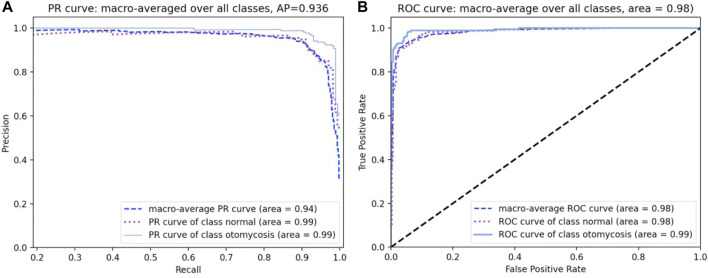
**(A,B)** Precision–recall and receiver operating characteristic curves of the four categories of otoendoscopic images.

The PR curve is used to sort the samples according to the predicted results of the classifier. The samples considered “most likely” to be positive by the classifier were in the front row, while those considered “least likely” to be positive by the classifier were in the back row. In this order, the samples were considered examples of positive prediction, and the current recall and precision were calculated each time. With accuracy as the vertical axis and recall as the horizontal axis, the PR curve was drawn. With the true-positive rate as the vertical axis and the false-positive rate as the horizontal axis, the ROC curve was drawn. Figures 5A,B show the results of the PR and ROC curves, respectively.

## 3 Results

We chose a deep CNN model to obtain a system that could help doctors accurately diagnose otomycosis. Three CNN models were selected for training, and the weighted mean of each model produced the highest verification accuracy.

To obtain better accuracy and generalization ability after model training, we selected 2,182 samples from approximately 4,000 otoendoscopic images as training samples and 475 samples as verification samples (Table 6). To select the deep CNN model, we tested the ResNet, SENet, and EfficientNet models with different layers. The optimal training results among the three models were selected. The average highest accuracies of ResNet101, SENet101, and EfficientNetB6 models were 78.32, 87.16, and 88.21%, respectively (Table 1). Considering the accuracy and speed of operation, we chose the EfficientNetB6 model to output the probability values of four types of otomycosis, impacted cerumen, external otitis, and the normal external auditory canal. After multiple iterative model training, the average accuracy of the overall validation sample was 92.42% (Table 3). The results suggest that the system could be used by doctors, or even patients, to better diagnose otomycosis.

**TABLE 6:** Data distribution in the classification test of otoendoscopic images.

Classification	Training set	Validation set	Test set	Total
Otomycosis	803	187	30	1,020
Impacted cerumen	264	61	30	355
External otitis	395	60	30	485
Normal case	720	167	30	917

We proposed a computer-aided decision system based on a deep learning model, which includes Java web application and image classification subsystems. The web application subsystem mainly provides a user-friendly page to collect images of consultation and display the calculation results. The image classification subsystem mainly uses a trained neural network model to perform end-to-end data reasoning. Finally, on uploading a few otoendoscopic images, the system returns the classification results to the user as the category probability value.

**FIGURE 6:**
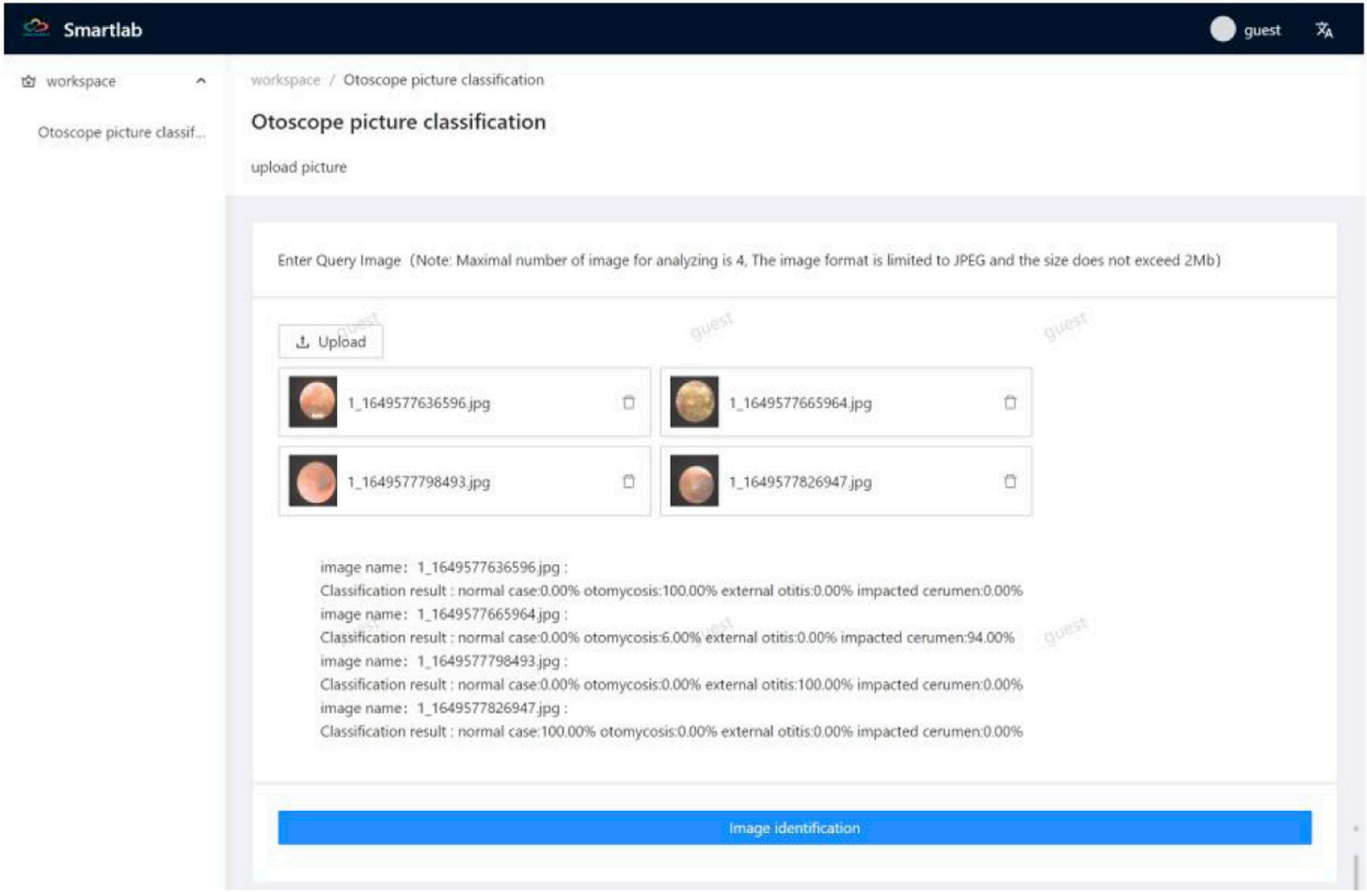
Screenshot of the authentication results on the webpage.

We released a beta web application at http://175.178.230.136/. Guests can login with a username and password and upload otoendoscopic images up to four at a time for diagnoses. The image size should not exceed 2 MB, and the image should be in the JPEG format. The “picture identification” button should be clicked to obtain probability values of the four categories. The uploaded image should be clicked to enlarge, read, and confirm the identification. Figure 6 shows the screenshot of the identification results on the website.

## 4 Discussion

Otomycosis is mainly diagnosed based on clinical manifestations and mycological examination results. However, differences in the pathogenic fungi species directly affects the positive rate of direct microscopic examination results (Ali et al., 2018). By culturing isolated specimens, further morphological and molecular biological identifications can be carried out, and the pathogenic species can be identified (Merad et al., 2021). However, conventional identification methods are time-consuming and prone to cross-contamination of specimens, which often leads to failure of clinicians to select effective antifungal drugs at a timely and early stage, affecting the prognosis (Hagiwara et al., 2019). Otoendoscopy has the advantages of a broad field of vision, close observation, and less invasive injury and has been widely used in the diagnosis and treatment of outer and middle ear diseases in recent years (Ulku, 2017). To treat otomycosis, we usually remove the fungal focus under ear endoscopy and select the appropriate drug according to the fungal culture results. Therefore, otoendoscopy and fungal culture are required to diagnose otomycosis. In our study, otoendoscopic images of impacted cerumen, external otitis, and the normal external auditory canal were used simultaneously. The established image library accumulated sufficient data to diagnose otomycosis by deep learning of otoendoscopic images.

We developed a computer-aided support system to assist physicians in the diagnosis of otomycosis. To ensure a diagnostic accuracy comparable to that of ear, nose, and throat specialists and provide the best care to patients, the most appropriate feature extraction methods and learning models were selected. A neural network model with different layers was tested, and the best training results of the three models was selected considering the accuracy and operation speed. The performance of the EfficientnetB6 model was found to be the highest. The weighted mean values of the models in the four categories were obtained. The result of each model was multiplied by the weighted mean value of each model and classification to obtain the average accuracy of the total validation sample of the set classifier (Table 1, Table 2, and Table 3). Classical machine learning techniques, such as SVM, K-NN, and decision tree, provide high performances in classification tasks, particularly with reasonably sized datasets (Van Gestel et al., 2002). These techniques are easy to understand, simplifying model tuning and calibration. Other more complex models, such as CNNs, can be used to overcome the same challenges but must be trained with larger database to achieve comparable performance.

The binary classification method of still color images of the eardrum has been used to identify the normal ear and otitis media, with accuracy rates of 73.1 and 68.3%, respectively (Tran et al., 2018; Cai et al., 2021). In both cases, color information was used to train the learning models. However, color alone cannot be used to obtain an accurate classification. In a previous study, classifying cases of normal ear and otitis media (Shie et al., 2014), the color, texture, and geometric information were used to train support vector machines with an accuracy exceeding 88.1% achieved by previous authors. However, the system’s specific ability to correctly identify healthy individuals was 79.9%. A study implemented a system to distinguish the normal eardrum, otitis media, and blocked ear canals with an accuracy of 86.8% (Myburgh et al., 2018). Whether these results were obtained through classification stages using validation or test sets remains unclear. The evaluation index of the deep CNN model selected in our study mainly depends on the performance of the set classifier, which is measured using the obfuscation matrix, precision, recall, and PR and ROC curves. The confusion matrix represents the number of instances corresponding to the predicted and actual classes (Table 4). To diagnose otomycosis, there were 187 images of otomycosis, 177, three, four, and three images of which were predicted to be of otomycosis, impacted cerumen, external otitis, and normal external auditory canal, respectively. Table 5 shows the statistics of the precision and recall. Figure 5 is drawn with the precision ratio as the vertical axis and the recall ratio as the horizontal axis to obtain the PR curve.

Previous studies on deep learning methods have distinguished between normal or abnormal conditions of the eardrum (Senaras et al., 2018). Two different deep learning architectures were used in this study, with an accuracy of 84.4%, a sensitivity of 85.9%, and a specificity of 82.8%. Another study proposed a diagnosis system based on deep CNN, with an average accuracy of 93.6% (Cha et al., 2019). The study divided ear diseases into five categories: invagination of the eardrum, perforation of the tympanum, tympanitis, external auditory canal tumors, and normal cases. However, the model performance, i.e., accuracy, depends on the number of images trained. If the number of images decreases, the performance degrades. A common limitation of deep learning approaches is the influence of database on the model (Zhang et al., 2020). In addition, a large database, particularly in otolaryngology, may be unavailable.

We introduced a novel beta web application, which is user-friendly. After several iterations of sample training, the average accuracy of the overall validation sample was 92.42%. Clinicians can login on the website and upload otoendoscopic images to accurately diagnose otomycosis (Figure 6). In the future, doctors and patients would be able to upload images to their smartphones or other devices to obtain diagnoses by installing software. The web application has a learning function. Therefore, the program can learn the uploading of identification images for improved accuracy.

To create a database of images of otomycosis, we collected otoendoscopic images of the external auditory canal obtained in clinical practice. According to the clinical manifestations and otoendoscopic images, otomycosis was considered, and specimens from these patients were collected for fungal species identification. The diagnostic system was relatively easy to implement and could significantly impact primary healthcare. Most blurred and unqualified images are deleted, and a few blurred images can also be analyzed and processed by artificial intelligence algorithms, thus increasing the diagnostic or classification accuracy. Although we randomly selected a large sample of cases, all possible imaging presentations of otomycosis may not have been covered. Therefore, image selection will have a potential bias. However, to avoid bias and improve accuracy, our database includes images of different tympanic membranes and external auditory canal projections.

Finally, we only evaluated four conditions that were presented during endoscopy, while the diagnosis of external auditory canal mycosis should include the differential diagnosis of other rare diseases in clinical practice. Whether our method would show reduced or improved accuracy if more conditions are included remains unknown. Therefore, the characteristics of otoendoscopy should be evaluated in detail in future studies. Nevertheless, compared to previous studies, our study achieved greater accuracy in the diagnosis of otomycosis in a real clinical setting through conditions that had not been previously assessed.

In future studies, we aim to integrate other rare types of otomycosis, although their diagnosis is a challenge even for specialists. In addition, we would train deep CNNs for other learning models, which may allow the integration of more classifications to maintain high performance.

## Data Availability

The original contributions presented in the study are included in the article/supplementary material, further inquiries can be directed to the corresponding author.
